# Dietary macroalgae enhances amino acid metabolism via intestinal *Shewanella* in grass carp (*Ctenopharyngodon idella*)

**DOI:** 10.1007/s44307-025-00090-8

**Published:** 2025-12-14

**Authors:** Xingxing An, Shuhui Niu, Mamun Abdullah Al, Erxin Su, Lin Chen, Houxiong He, Yaohua Wang, Song Zhang, Yong Yang, Shen Wang, Zheyu Wen, Baohong Xu, Yuzhen Ming, Wengen Zhu, Zhenrui Zhao, Kun Wu, Yufeng Yang, Wei Xie, Zhili He, Qingyun Yan

**Affiliations:** 1 School of Marine Sciences, Marine Synthetic Ecology Research Center, Southern Marine Science and Engineering Guangdong Laboratory (Zhuhai), Sun Yat-sen University, Zhuhai, 519082 China; 2 Guangzhou Nutriera Biotechnology Co., Ltd., and Guangdong Nutriera Group Co., Ltd, Zhuhai, 519100 China; 3 Zhuhai Haifa MarineHarvest Industrial Park Co., Ltd, Zhuhai, 519006 China; 4 College of Aquaculture, Hunan Agricultural University, Changsha, 410128 China

**Keywords:** Amino acid metabolism, Grass carp (*Ctenopharyngodon idella*), Gut microbiome, Macroalgae, *Shewanella*

## Abstract

The gut microbiome plays pivotal roles in the host’s metabolic response to dietary interventions. Dietary macroalgae supplementation represents a promising strategy for enhancing animal growth and health via microbiome modulation. However, the underlying mechanism of how macroalgae supplementation regulates microbiome-host interactions in aquatic species remains unclear. This study investigated the effects of three dietary macroalgae—*Sargassum hemiphyllum* (S), *Asparagopsis taxiformis* (A), and *Gracilaria lemaneiformis* (G)—each supplemented at 5% in feed, on the gut microbiome and metabolism of grass carp (*Ctenopharyngodon idella*), using integrated approaches of 16S rRNA sequencing, metagenomics, and metabolomics. While all three macroalgae influenced host growth, supplementation of S provided the most comprehensive benefits, with significant enhancement of body weight and hepatic superoxide dismutase activity. Integrated multi-omics analysis revealed that dietary macroalgae supplementation increased the relative abundance of the key gut bacterial genus *Shewanella*, with the most notable effect observed in the supplementation of S. Subsequent analysis of a metagenome-assembled genome (MAG) of *Shewanella* (MAG C3_bin52) demonstrated its considerable potential for amino acid biosynthesis and metabolism. This genomic potential was further supported by metabolomic profiling, which indicated significant upregulation of amino acid-related metabolites, particularly in the supplementation S. Pathway analysis confirmed enrichment in processes associated with protein digestion and absorption, amino acid biosynthesis, and related metabolic pathways. These findings highlight the modulation of a macroalgae-microbiome-metabolite axis in grass carp, primarily mediated by the enrichment of *Shewanella* in gut ecosystem for enhancing host amino acid metabolism. This study advances understanding of dietary modulation of the gut microbiome and provides insights for the sustainable development of aquaculture.

**Supplementary Information:**

The online version contains supplementary material available at 10.1007/s44307-025-00090-8.

## Introduction

The gut microbiome, a complex microbial ecosystem within the animal gut, significantly affects host growth, immunity and metabolism (Ma and Ma., 2019; Zhuang et al. 2025; Zeng et al., 2020). Shifts in its composition can alter host metabolic pathways, highlighting the important interactions between the gut microbiome and host metabolic processes (Bogatyrev et al. 2020; Wu et al. 2022). Previous studies indicate that gut microbiome modulates lipid metabolism in fish via mechanisms involving lipases and short-chain fatty acids (Wu et al. 2024). Specifically, *Shewanella* species in gut can directly influence host metabolic states via specialized pathways. For example, dietary supplementation with the probiotic *Shewanella putrefaciens* (SpPdp11) has been demonstrated to enhance growth performance and disease resistance in fish by improving host metabolism and immunity (Cámara-Ruiz et al. 2020). Strains like *Shewanella* sp. N2AIL, isolated from fish gut, can biosynthesize health-promoting ω−3 polyunsaturated fatty acids (Chaudhary et al. 2022), which are vital for anti-inflammatory responses and neural development in aquatic species.

Diet is a primary determinant of gut microbiome composition and diversity, influencing host metabolism and health through host-microbiome interactions (Montoya-Ciriaco et al. 2020; David et al. 2014; Schoeler et al. 2023). For instance, diets enriched with long-chain unsaturated fatty acids modulate gut microbial communities and reduce hepatic steatosis in mice (Schoeler et al. 2023). Similarly, changes in dietary protein sources alter gut microbiota composition and diversity in rainbow trout (Pérez-Pascual et al. 2021). Beyond macronutrients, specific functional feed additives also mediate their effects via the gut microbiota. For example, dietary butyrate supplementation has been shown to improve immune function and metabolic status in fish by regulating the gut microbiota (Piazzon et al. 2017). Also, the inclusion of algal polysaccharides in feed has been reported to enhance the physiological health of fish by boosting the activity of antioxidant enzymes (Huang et al. 2025). These findings suggest that dietary components can strategically modulate gut microbiota.

Macroalgae, rich in various nutrients and bioactive compounds, are valuable dietary supplements in aquaculture (Harsha Mohan et al. 2023; Peñalver et al. 2020; Tanna and Mishra 2018). In particular, *Sargassum hemiphyllum*, *Asparagopsis taxiformis*, and *Gracilaria lemaneiformis* have attracted significant research interest due to their functional properties such as immunomodulatory, antioxidant, and gut-health-promoting effects (Li et al. 2022; Pereira et al. 2024; Bakky et al. 2023). *S. hemiphyllum* is characterized by its sulfated polysaccharides, which exhibit notable immunomodulatory and anti-inflammatory activities (Hwang et al. 2011; Li et al. 2022). *A. taxiformis* is recognized for its antioxidant capacity and antimicrobial efficacy against fish pathogens, which can enhance physiological resilience under stress (Marino et al. 2016; Pereira et al. 2024). *G. lemaneiformis* and its derived polysaccharides have been shown to improve gut health by modulating microbial community and stimulating the production of beneficial short-chain fatty acids (Bakky et al. 2023; Lu et al. 2021). Despite these well-documented benefits of macroalgae effects on animal growth and immunity, the mechanisms by which macroalgae-induced shifts in the gut microbiota mediate metabolic responses in aquatic species remain unclear.

We hypothesize that dietary macroalgae supplementation, via their rich amino acids and polysaccharides, selectively enriches microbial taxa (e.g., *Shewanella*) with specialized metabolic functions to utilize these substrates. *Shewanella* possesses versatile metabolic capabilities, including pyruvate/lactate metabolism (Pinchuk et al. 2011), complex polysaccharides degradation (Yagi et al. 2018), and amino acid transport/biosynthesis (Chaudhary et al. 2022). This microbiome remodeling would drive functional adaptations in amino acid biosynthesis and metabolism pathways, thereby enhancing host nutrient utilization, growth performance, and metabolic homeostasis.

The grass carp (*Ctenopharyngodon idella*) was selected as the model species to test this hypothesis, based on its critical economic importance in global freshwater aquaculture (FAO 2024) and its herbivorous habit, which is anticipated to facilitate the utilization of macroalgae. Using 16S rRNA gene sequencing, we examined the impact of three macroalgae diets on its gut microbiome. We further integrated metagenomics and metabolomics to explore linkages between microbiome functional potential and metabolite profiles. This study evaluates the effects and mechanisms of dietary macroalgae supplementation in grass carp, providing new insights into how macroalgae modulate host metabolism and health through gut microbiota.

## Methods

### Macroalgae supplementation in feeds

To investigate macroalgae supplementation effects on aquaculture species and elucidate underlying mechanisms, we selected three species of macroalgae: *Sargassum hemiphyllum* (S), *Asparagopsis taxiformis* (A), and *Gracilaria lemaneiformi*s (G). A control diet only replaced the 5% macroalgae component with bean oil. *S. hemiphyllum* and *G. lemaneiformis* were obtained from Shandong province, while *A. taxiformis* was collected from Hainan province, China. Collected fresh macroalgae samples were washed thoroughly to remove surface contaminants, air-dried and ground into powders using a laboratory mill. All other ingredients were similarly ground with a mechanical grinder and sieved through an 80-mesh screen. The prepared mixtures were subsequently extruded into 2.0 mm pellets using a laboratory-scale pelletizer, and then dried at 75 °C. All four iso-nitrogenous and iso-lipidic diets differed solely with the 5% macroalgae powders supplementation. Detailed ingredients and chemical compositions of these diets are presented in Tables S1 and S2.

### Experimental design and fish husbandry

Grass carp were maintained in a recirculating aquaculture system at Guangdong Nutriera Biotechnology Co., Ltd. (Zhuhai, China) for 52 days. Water temperature was kept at 25–28 °C during the experiment. After a week’s acclimation, healthy fish (91.50 ± 0.66 g) were randomly allocated to four dietary treatment groups, which were given the feeds S, A, G, and Control, respectively. Each group contained three replicated buckets (radius = 0.4 m, height = 1.5 m, 30 fish per bucket). The fish were fed to apparent satiation twice daily (9:00 and 16:00), with no mortality occurring throughout the experiment. Residual feed and waste were removed after each feeding session. Water quality was maintained through daily partial water exchanges.

### Sample collection

After a 52-day rearing period, fish were fasted 24 h prior to sampling. Six individuals per dietary group (two from each bucket) were randomly selected, anesthetized with tricaine methanesulfonate (MS-222, 100 mg/L). The body length and weight were measured directly, blood of each fish was collected from the caudal vein and centrifuged at 3500 rpm for 15 min to obtain serum. Fish were dissected to obtain viscera and liver samples. The viscerosomatic index (VSI) and hepatosomatic index (HSI) were determined according to the following formulas: VSI (%) = (viscera weight/body weight) × 100, and HSI (%) = (liver weight/body weight) × 100. For gut microbiome analysis, the entire intestine was aseptically removed (three fish per group). All collected samples were flash-frozen in liquid nitrogen and then stored at −80 °C for subsequent analysis.

For collecting fecal samples in subsequent metagenomics and metabolomics analysis, 12 independent sterile systems were established. Glass tanks (length × width × height = 70 × 40 × 40 cm), aeration stones, and tubing were disinfected with sodium hypochlorite solution, rinsed extensively with ultrapure water, and UV-sterilized (1–3 h). After 24 h of fasting, three fish were randomly selected from each bucket were correspondingly transferred to one sterile system. Freshly excreted feces in each tank were immediately collected using sterile pipettes, placed in pre-chilled sterile centrifuge tubes, and stored at −80 °C for subsequent analysis.

### Physiological and biochemical analysis

Serum samples were analyzed to determine lysozyme (LZM) and total cholesterol (TC) concentrations using kits for lysozyme assay (A050-1) and total cholesterol assay (A111-2), respectively. Hepatic tissues (0.2 g per sample) were homogenized and ground with 1.8 mL of 0.88% NaCl solution. The homogenate was centrifuged at 2000 × g at 4 °C for 15 min, and the supernatant was collected to measure hepatic protein concentration using a total protein assay kit (A045-4) based on the bicinchoninic acid (BCA) method. Hepatic superoxide dismutase (SOD) activity was assessed using an SOD assay kit (1001–2) according to the method as described by the manufacturer. All the kits used herein were ordered from the Nanjing Jiancheng Bioengineering Institute.

### 16S rRNA gene sequencing analysis

The collected intestinal sample of each fish was homogenized to extract DNA using a PowerFecal® DNA isolation kit (Mo Bio Laboratories, CA, USA) following manufacturer's instructions. DNA concentration and quality were determined using a NanoDrop-2000 spectrophotometer (Thermo Fisher Scientific, MA, USA). The V3-V4 hypervariable regions of the 16S rRNA gene were amplified using the primers 341 F (5'-CCTAYGGGRBGCASCAG-3') and 806R (5'-GGACTACNNGGGTATCTAAT-3'). The amplified products were sequenced on an Illumina HiSeq 3000 platform at Major Bio. Raw reads underwent quality filtering and merging in QIIME2 (Bolyen et al. 2019) (v2023.5). The sequences were denoised and assembled into amplicon sequence variants (ASVs) using DADA2(Callahan et al. 2016). 107 ASVs were retained for further downstream analysis after removing those with an average relative abundance of less than 0.01% and occurrences in fewer than 0.2 of the total sample size. Representative sequences were assigned taxonomy using the Silva reference database (v138, 99% similarity threshold) (Yilmaz et al., 2014). Using the Random Forest method (Breiman, 2001), we further identified significant microbiome features at the genus level across different groups. Subsequently, significant differences in bacterial genera abundance between groups were identified through Dunn’s test for multiple comparisons, with *p*-values adjusted via the Benjamini–Hochberg method to control the false discovery rate (FDR < 0.05).

### Metagenome sequencing analysis

DNA was extracted from fecal samples using the PowerFecal® DNA isolation kit (Mo Bio Laboratories, CA, USA). Extracted DNA from 12 fecal samples underwent end-repair, adapter ligation, and high-throughput sequencing on Illumina Novaseq6000 platform, yielding 392.32 Gb of raw data (> 30 Gb per sample). Quality control was performed using the FastQC (Brown et al 2017), and low-quality bases and adapters were removed using the BBDuk (quality score > 20, length > 70 bp). Quality-filtered reads were de novo assembly into contigs using MEGAHIT (v1.1.2) (Li et al. 2015). Taxonomic and functional traits of contigs were annotated using Kraken2 (v2.0.9) (Wood et al. 2019) and DIAMOND (Buchfink et al 2015), respectively. Contigs were then binned into metagenome-assembled genomes (MAGs) with Metabat2 (v2.12.1) (Kang et al. 2015), followed by refinement using metaWRAP pipeline (v1.3.2), (consensus score > 50%). MAG quality was assessed with CheckM (v1.0.12) (Parks et al. 2015) to obtain high-quality MAGs (completeness > 90% and contamination < 5%). Taxonomic classification of MAGs was performed using Kraken2 (v2.0.8-beta) through the highest-scoring assignments (Wood et al. 2019). Functional annotation involved open reading frame (ORF) prediction via Prodigal (v2.6.3) (Hyatt et al 2010) in meta mode, and non-redundant gene catalogs construction using CD-HIT v4.8.1 (95% nucleotide identity, 90% coverage) (Huang et al 2010). Gene abundance was quantified with Salmon (v0.13.0) in alignment-based mode and normalized as transcripts per million (TPM) (Patro et al. 2017). KEGG pathways were annotated through KofamKOALA (v1.3.0) (Aramaki et al. 2020) with default threshold, and metabolic pathways were reconstructed via METABOLIC (v4.0) (Zhou et al. 2022).

### Metabolomics analysis

Fecal samples (50 mg per sample) were homogenized with 400 μL ice-cold methanol/water (4:1, v/v) containing 0.02 mg/mL L-2-chlorophenylalanine (internal standard) using cryogenic grinding (−10 °C, 50 Hz, 6 min) followed by ultrasonic extraction (5 °C, 40 kHz, 30 min). After an incubation (−20 °C, 30 min) and centrifugation (13,000 × g, 4 °C, 15 min), supernatants were analyzed using UHPLC-Q Exactive HF-X system (Thermo Fisher Scientific, MA, USA) with Hydrophilic Interaction Liquid Chromatography (HILIC) chromatography. Raw Liquid Chromatography-Mass Spectrometry (LC–MS) data were processed through Progenesis QI(Deutsch et al. 2015) (Waters) for feature extraction, alignment, and HMDB annotation (http://www.hmdb.ca/). Data matrices were filtered by: (1) retaining features present in > 80% samples of each group; (2) filling missing values with half-minimum; (3) applying total sum normalization; (4) removing QC features with RSD > 30%; (5) performing log_10_-transformation. Differential metabolites (VIP > 1, *p* < 0.05) identified through partial least squares-discriminant analysis (PLS-DA) (Barker and Rayens 2003) via *ropls* R package (sevenfold cross-validation) were functionally annotated using KEGG pathway enrichment. All metabolomics analyses were performed on the Majorbio Cloud Platform (https://www.majorbio.com/web/www/index) (Han et al. 2024).

### Statistical analysis

All statistical analyses were performed using GraphPad Prism (v9.5, GraphPad Software, CA, USA). Normality of data was assessed using Shapiro–Wilk test. Comparisons between two groups with normal distribution were analyzed using Student’s *t*-test. The Kruskal–Wallis test was applied for multi-group comparisons involving non-parametric data, followed by Dunn's test performed with the Benjamini–Hochberg correction. Significant threshold was set as *p* < 0.05. Microbiome-metabolite relations were assessed via Spearman rank correlation (|*ρ*|> 0.6; Benjamini-Hochberg-adjusted *p* < 0.05). Relationships among *Shewanella* abundance, amino acid metabolites, and fish indicators were modeled via Partial Least Squares Path Modeling (PLS-PM) using R packages of *plspm* and *dplyr*. The model incorporated multiple latent variables, including relative abundances of gut microbiota, amino acid metabolites, growth and immune indicators. Model reliability was assessed using the goodness-of-fit (GOF) metric.

## Results

### Effects of dietary macroalgae on growth and immunity of grass carp

**Fig. 1:**
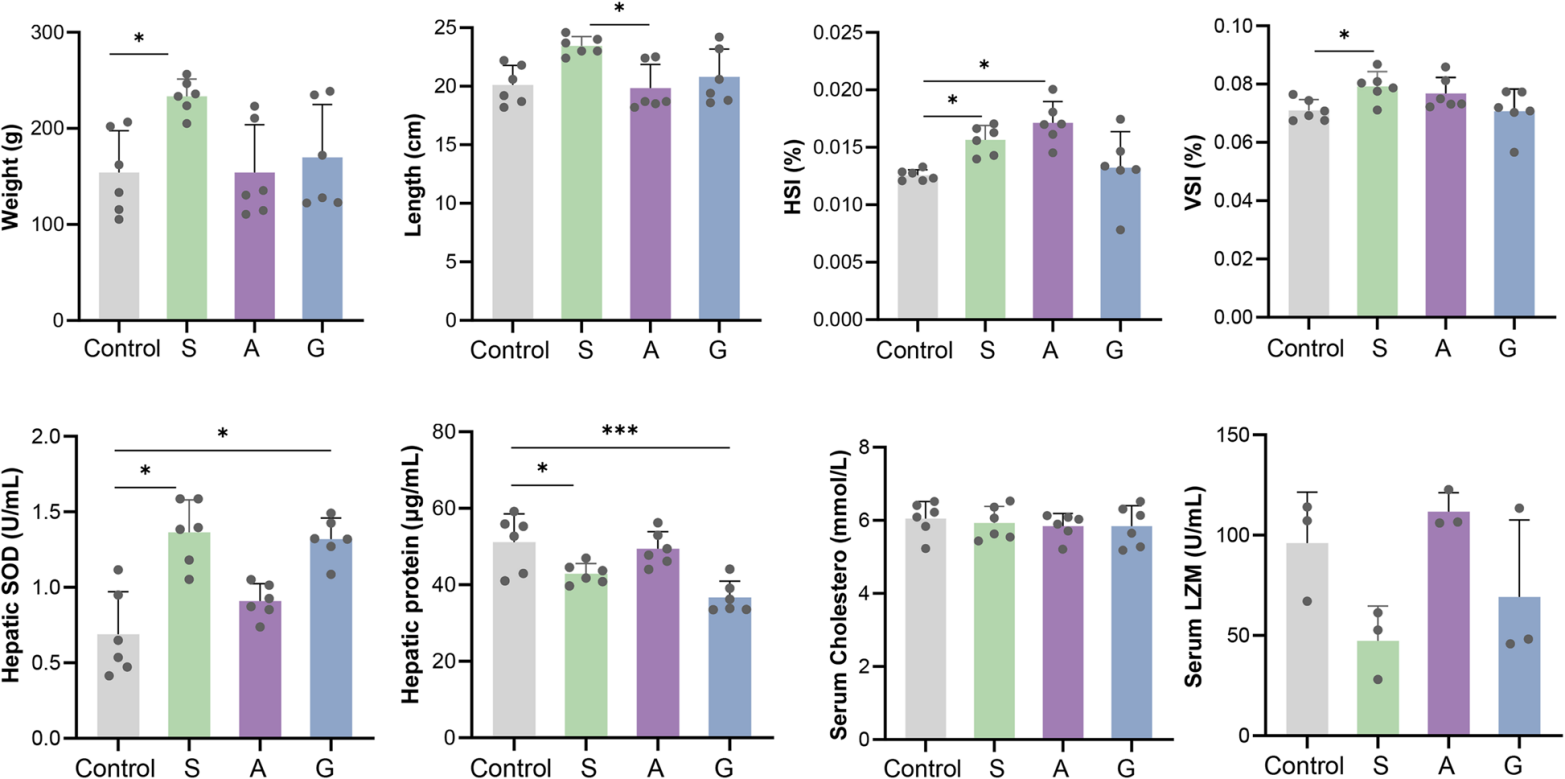
Physiological and biochemical responses of grass carp to dietary macroalgae supplementation. Macroalgae diets included S (5% *Sargassum hemiphyllum*), A (5% *Asparagopsis taxiformis*), and G (5% *Gracilaria lemaneiformis*). HSI: hepatosomatic index; VSI: viscerosomatic index; SOD: superoxide dismutase; LZM: lysozyme. Statistical significance: * *p* < 0.05, ** *p* < 0.01, *** *p* < 0.001

Macroalgae supplementation significantly affected the growth performance and immunity of grass carp (Fig. 1). Group S exhibited significantly greater body weight, VSI and HSI than that of the control (*p* < 0.05). Group A showed significantly greater body length and VSI than that of the control (*p* < 0.05). Immunologically, hepatic protein concentration decreased significantly in groups S and G, while group S displayed significant enhancement of hepatic SOD activity (*p* < 0.05). These results demonstrate that dietary macroalgae, especially the supplementation of *S. hemiphyllum*, provides the most comprehensive benefits for growth enhancement and antioxidant activity in grass carp.

### Effects of dietary macroalgae on gut microbiome composition

**Fig. 2:**
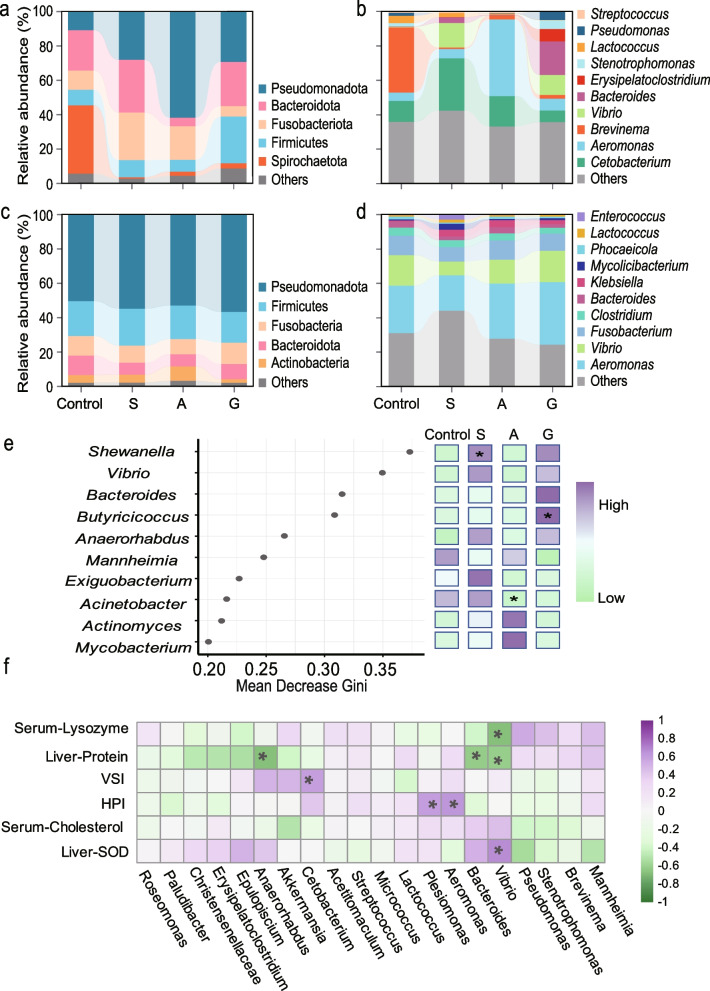
Impact of dietary macroalgae supplementation on the intestinal microbiome composition of grass carp. **a**, **b** Taxonomic composition at the phylum (top five) phyla and genus (top ten) levels based on 16S rRNA gene sequencing of intestinal samples. **c**, **d** Corresponding taxonomic profiles derived from metagenome sequencing of fecal samples. **e** Random Forest analysis identifying the top ten discriminant intestinal genera between treatment groups. The y-axis presents the ranking of bacterial genera based on their importance scores, while the x-axis denotes the Mean Decrease in Accuracy. The right panel displays a heatmap illustrating the abundance profiles of these genera across groups, with data standardized through z-score normalization. Asterisks indicate significant differences (Dunn's test with Bonferroni correction, *p* < 0.05). **f** Spearman correlation heatmap between immune parameters and intestinal bacteria abundances. Macroalgae diets included S (5% *Sargassum hemiphyllum*), A (5% *Asparagopsis taxiformis*), and G (5% *Gracilaria lemaneiformis*)

The 16S rRNA gene sequencing of intestinal samples revealed that Spirochaetota was the predominant phylum (37.79%) in the control group, followed by Bacteroidota (23.60%), Fusobacteria (12.28%), and Pseudomonadota (11.81%) (Fig. 2a). In contrast, dietary macroalgae supplementation significantly increased the relative abundance of Pseudomonadota to 28.08% (group S), 61.83% (group A), and 29.34% (group G), while drastically reducing Spirochaetota to < 0.03%. At the genus level (Fig. 2b), B*revinema* dominated the control group (37.79%) but decreased in all macroalgae-supplemented groups. The most abundant genera in groups S, A, and G were *Cetobacterium*, *Aeromonas*, and *Bacteroides*, respectively. Metagenome sequencing of fecal samples confirmed this microbial composition pattern in the intestine, with Pseudomonadota, Firmicutes, Fusobacteria, and Bacteroidota as the dominant phyla (Fig. 2c). At the genus level, group S showed lower relative abundances of *Aeromonas* and *Vibrio* compared to the control, whereas groups A and G exhibited similar compositions to the control (Fig. 2d).

To identify key bacterial genera associated with macroalgae supplementation, we performed feature selection using a Random Forest model (Fig. 2e) and conducted differential abundance analysis at the genus-level using the Dunn test (Table S3). The results revealed *Shewanella* as the most significant genus (Fig. 2e), with a significant higher abundance in group S compared to the control. Additionally, *Vibrio*, *Bacteroides*, and *Butyricicoccus* were identified as potentially important genera. Specifically, *Butyricicoccus* abundance was significantly increased in group G compared to the control, whereas *Acinetobacter* was significantly lower in group A.

Furthermore, correlation analysis demonstrated that *Vibrio* abundance was positively correlated with hepatic SOD activity (r = 0.66, *p* < 0.05) but negatively correlated with serum LZM activity (r = −0.68, *p* < 0.05) and total hepatic protein content (*r* = −0.62, *p* < 0.01). *Aeromonas* showed a strong positive correlation with the HSI (r = 0.64, *p* < 0.05), whereas *Cetobacterium* showed a moderate association with the VSI (r = 0.60, *p* < 0.05) (Fig. 2f). These findings suggest that macroalgae modulates the gut microbiota network and influences growth-immune parameters in grass carp, highlighting the complex relationships between gut microbiome and host physiology.

### Effects of dietary macroalgae on gut microbiome functional potentials

Metagenome sequencing of fecal samples revealed significant alterations in functional gene abundances with dietary macroalgae supplementation. Notable differences in KEGG Orthologs (KOs) were observed between macroalgae and control groups (|log_2_ (Fold Change) |> 1 and *p* < 0.05). Macroalgae supplementation of S and A showed greater improvement in amino acid and carbohydrate metabolism (Figures S1b, S1c), while the supplementation of G mainly enhanced terpenoid, polyketide, and lipid metabolism (Figure S1d).

Contigs analysis demonstrated that macroalgae supplementation significantly increased the abundance of functional genes associated with amino acid metabolism (Figure S2). Specifically, group S exhibited increased abundances of *ADT/PDT* (tyrosine/tryptophan biosynthesis) and *astE* (arginine/proline metabolism), genes compared to that of the control (*p* < 0.05). In contrast, group A showed reduced *lysC* and *adiA* gene abundances (*p* < 0.05), while group G displayed increased *tyrB* and *metY* gene abundances compared to that of the control (Figure S2).

**Fig. 3:**
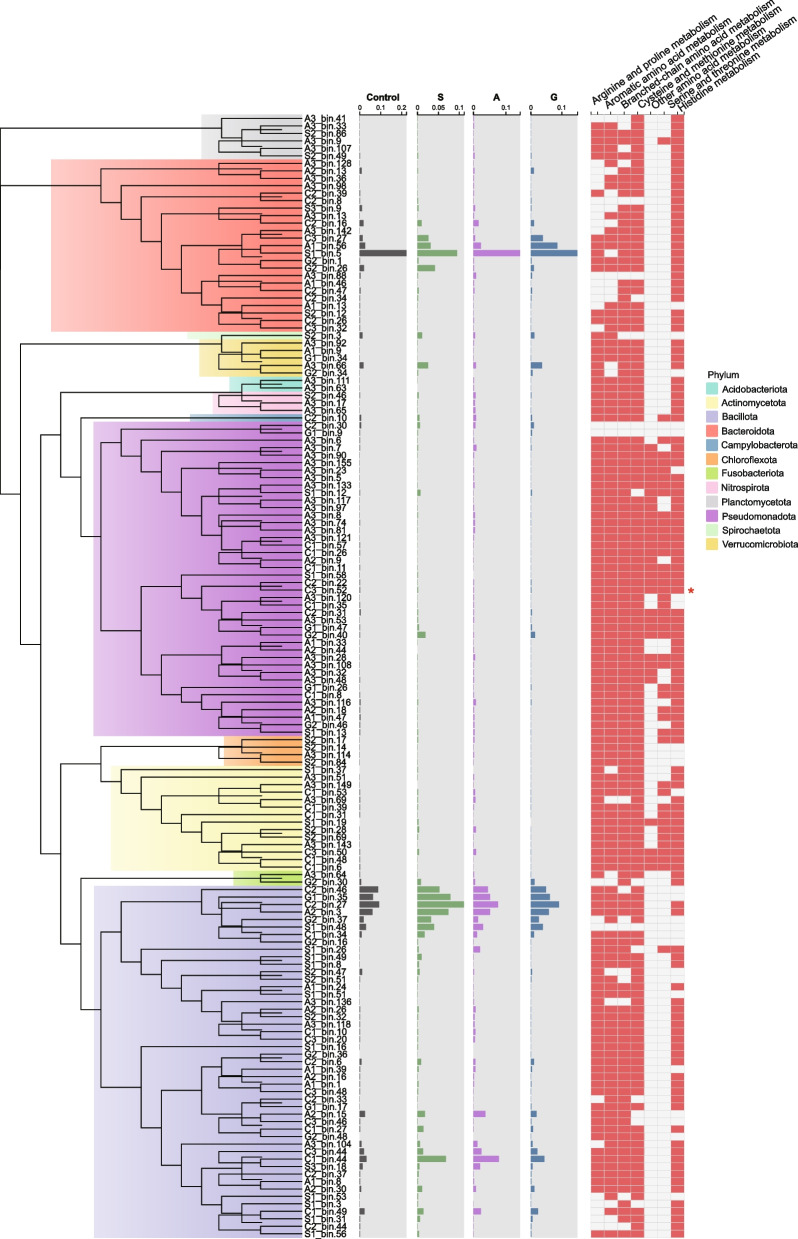
Phylogenetic analysis and distribution of high-quality metagenome-assembled genomes (MAGs) recovered from grass carp fecal samples. The bar chart (middle panel) shows the relative abundance of each MAG. The presence (red) and absence (blank) of protein-encoding genes (right panel), where the asterisk (*) indicates MAG C3_bin52. Macroalgae diets included S (5% *Sargassum hemiphyllum*), A (5% *Asparagopsis taxiformis*), and G (5% *Gracilaria lemaneiformis*)

To investigate the microbial metabolic roles, we recovered 150 high-quality MAGs (Bacillota 47, Pseudomonadota 42, Fig. 3). Functional annotation revealed their strong involvement in core metabolic pathways, particularly amino acid and carbohydrate processing. Pseudomonas MAGs were the predominant carriers of serine and threonine metabolism genes (64% and 71% pathway prevalence). The MAG C3_bin52 (*Shewanella*) harbored diverse amino acid metabolism genes, consistent with its increased abundance in macroalgae supplementation groups (Fig. 2). Metabolic profiling of C3_bin52 identified 2,488 KOs, with 10.29% associated with amino acid metabolism (Figure S3).

### Effects of dietary macroalgae on metabolism in grass carp

**Fig. 4:**
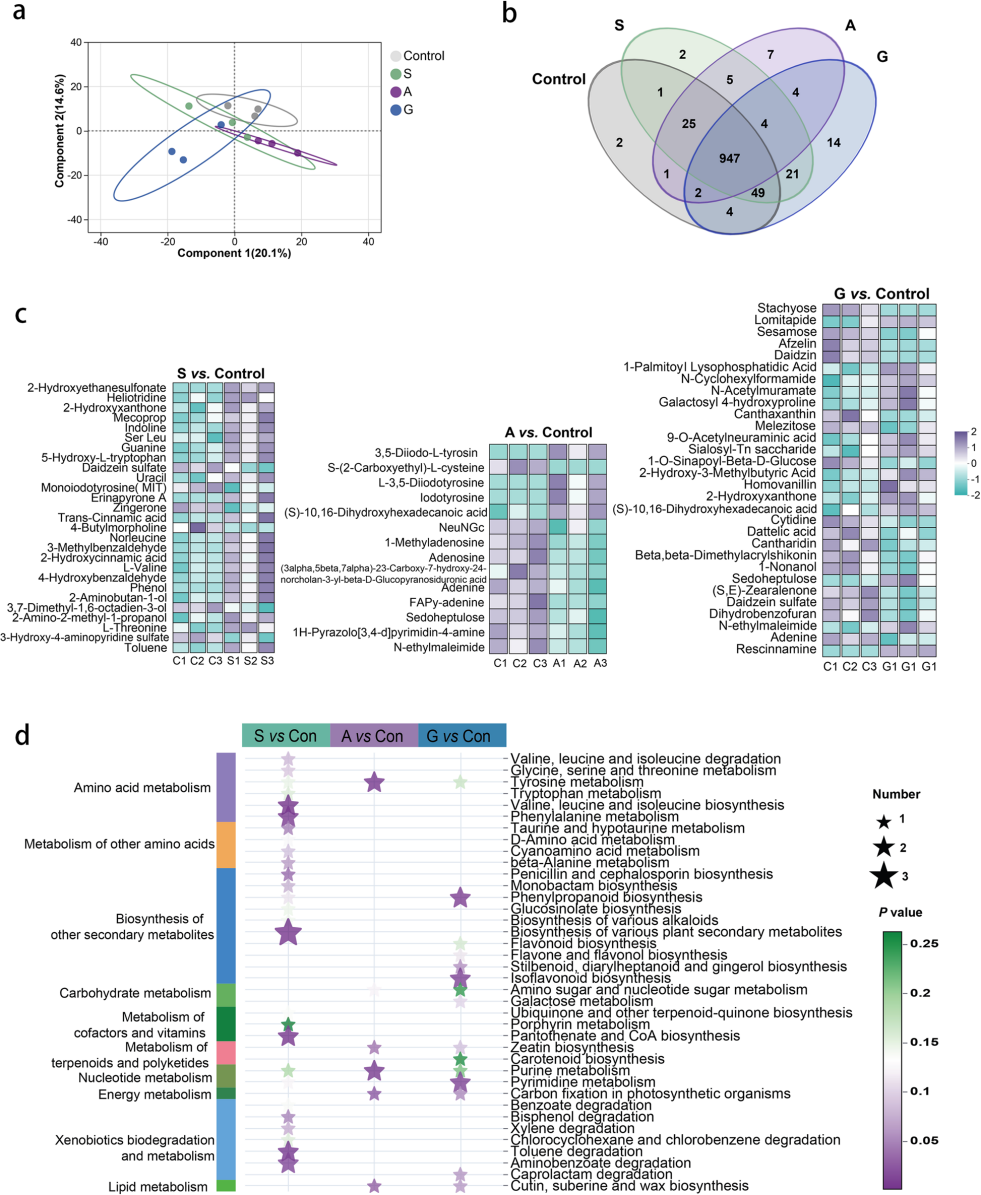
Metabolic proofing of grass carp influenced by dietary macroalgae supplementation. **a** Partial least squares-discriminant analysis (PLS-DA) plot demonstrating group separation of fecal metabolomes (95% confidence ellipses shown). **b** Venn diagram showing shared and unique metabolites. **c** Differential abundance heatmap of significantly altered metabolites between control and macroalgae groups. **d** KEGG pathway enrichment analysis highlighting metabolic pathways affected by macroalgae supplementation. Macroalgae diets included S (5% *Sargassum hemiphyllum*), A (5% *Asparagopsis taxiformis*), and G (5% *Gracilaria lemaneiformis*). C/Con: control diet

To elucidate the interactions between dietary macroalgae, gut microbiome, and host metabolism, we conducted non-targeted metabolomics profiling of fecal samples. PLS-DA score plot showed clear separation of metabolites among groups (Fig. 4a), with the first two principal components explained 34.70% of total variance. Metabolite distribution analysis identified 947 metabolites, including two unique metabolites to the control and group S, 7 and 14 unique to groups A and G, respectively (Fig. 4b). Differential metabolites analysis demonstrated group-specific metabolic responses (Fig. 4c). Group S showed 21 up-regulated metabolites, primarily amino acids (L-valine, L-threonine, leucine) and their derivatives (5-hydroxy-L-tryptophan, trans-cinnamic acid). Group A exhibited overall metabolite down-regulation but significant up-regulation of tyrosine metabolites (L-3,5-diiodotyrosine, iodotyrosine). Group G exhibited 14 up- and 25 down-regulated metabolites, including decreased purines (adenine, cytosine) and increased homovanillin (tyrosine metabolites).

Metabolic pathway enrichment analysis revealed distinct functional patterns for macroalgae groups (Fig. 4d). Group S primarily enriched in protein digestion and absorption, amino acid biosynthesis, mineral homeostasis, and heterometabolite degradation pathways. Group A strongly associated with thyroid hormone synthesis, tyrosine metabolism, and purine-nucleotide metabolic networks. Group G mainly enriched in phenylpropanoid biosynthesis and nucleotide metabolic pathways. These results demonstrate that macroalgae supplementation differentially modulates fish metabolism: groups S and A primarily influence valine and tyrosine respectively, while group G predominantly affects nucleotide metabolism.

### Correlation between gut microbiome and metabolites

**Fig. 5:**
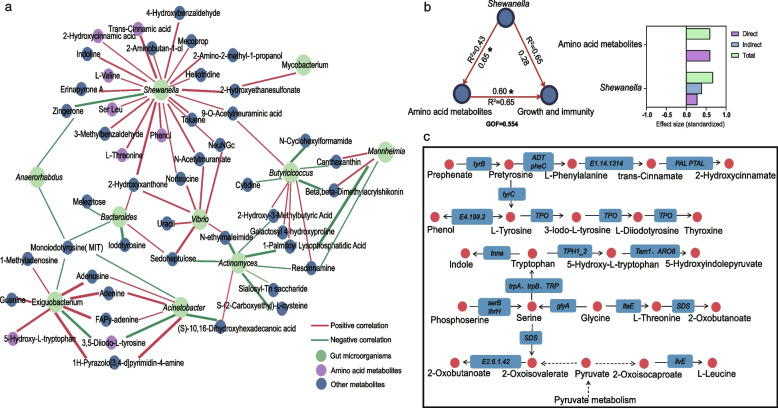
Intestinal microbiome-metabolite associations in grass carp. **a** Correlation network between intestinal microbiome and metabolites. Line thickness corresponds correlation strength (only |r|> 0.6 and *p* < *0.05* are given). **b** Partial least squares-structural equation modeling (PLS-PM) illustrating relationships among *Shewanella* abundance, amino acid metabolites, and grass carp performance. Red arrows indicate positive path coefficients (values shown), with asterisks indicating statistical significance (* *p* < 0.05). The goodness-of-fit (GOF) reflects the overall model fit. **c** Proposed framework for *Shewanella*-mediated modulation of amino acid metabolic pathways derived from multi-omics integration

Our integrated multi-omics analysis revealed a robust correlation network between gut microbiome and host metabolism (Fig. 5a). Spearman correlation analysis identified 78 significant microbiome-metabolite associations (|r|> 0.6, *p* < 0.05), with *Shewanella* emerging as the central hub with the highest connectivity. *Shewanella* displayed positive correlations with 22 metabolites, particularly aromatic amino acid derivatives (2-hydroxycinnamic acid, r = 0.63) and branched-chain amino acids (L-valine, r = 0.63). *Exiguobacterium* and *Acinetobacter* co-varied with six metabolites, including thyroid hormone precursors (5-diiodo-L-tyrosine). *Vibrio* showed a dichotomous association (positive with six metabolites but negative with monoiodotyrosine, r = −0.83). *Butyricicoccus* correlated positively with N-cyclohexylformamide (r = 0.70) and seven other metabolites, while negatively correlated with three metabolites. Bacteroides demonstrated negative correlation with iodotyrosine (r = −0.73).

**Fig. 6:**
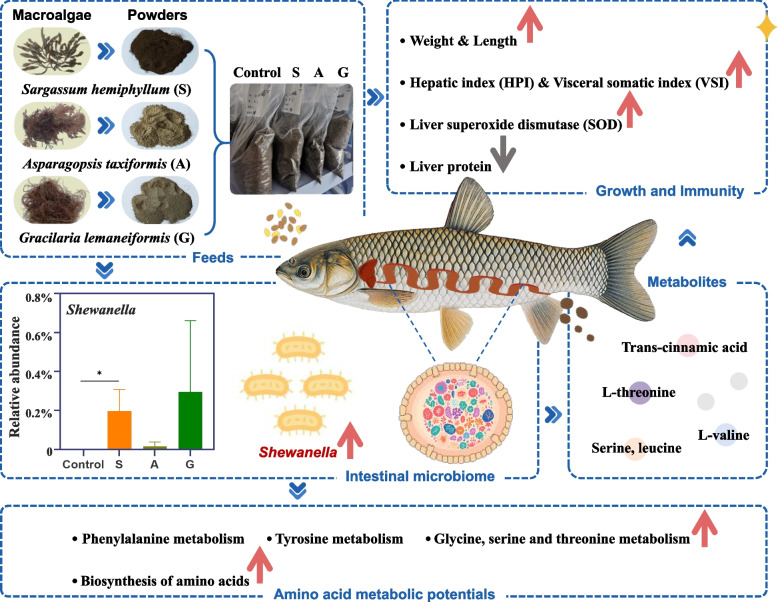
Proposed conceptual model of dietary macroalgae-mediated regulation of amino acid metabolism in grass carp via intestinal microbiome modulation. The red upward arrow represents an increase, while the gray downward arrow represents a decrease

To validate *Shewanella*’s role in amino acid metabolism, we performed PLS-PM (Fig. 5b). The model demonstrated moderate explanatory power (goodness-of-fit = 0.554). We observed a positive association between amino acid metabolites and growth performance (coefficient = 0.596, *p* < 0.05). *Shewanella’s* abundance was positively correlated with amino acid metabolism (coefficient = 0.655, p < 0.05). KEGG pathway reconstruction (Fig. 5c) confirmed *Shewanella*’s metabolic potentials in mediating the metabolism and synthesis of amino acids (valine, threonine). These processes could be modulated by *Shewanella*’s key genes encoding branched-chain amino acid transferase (e.g., *ilvE*) and amino acid synthesis genes (e.g., *trpA*, *trpB*, and *SDS*). These findings collectively demonstrate that *Shewanella* enhances grass carp growth through modulation of amino acid metabolic pathways, explaining the significant (*p* < 0.05) changes of amino acids metabolites observed in macroalgae supplemented groups (Fig. 6).

## Discussion

The gut microbiome is a key regulator of host metabolism (Wu et al. 2022, 2024; Murga-Garrido et al. 2021), and dietary macroalgae supplementation alters gut microbiota and enhances host growth (Peñalver et al. 2020; Siddik et al. 2023). However, interactions between macroalgae supplementation, gut microbiome, and host metabolic reprogramming in aquatic animals remain poorly understood. Our study addresses this gap by demonstrating that macroalgae supplementation promotes amino acid metabolism in grass carp through enrichment of *Shewanella*.

The core gut microbiota of grass carp—dominated by Bacteroidota, Fusobacteria, Pseudomonadota, and Firmicutes—significantly contributes to host metabolism and health (Li et al. 2024). Dietary macroalgae provide abundant polysaccharides and free amino acids (Harsha Mohan et al. 2023; Peñalver et al. 2020; Tanna & Mishra, 2018), which are enzymatically depolymerized into absorbable metabolites by gut microbiota (Hong et al. 2011). This substrate shift drove selective enrichment of polysaccharolytic guilds such as *Shewanella*, which exhibits strong anaerobic metabolic capabilities (Lemaire et al. 2020), efficiently degrades polysaccharide and utilizes diverse organic substrates (Wang et al. 2021). Interestingly, *Shewanella* abundance increased significantly in group S than that in the control, indicating targeted microbial adaptation to dietary macroalgae. Crucially, *Shewanella* enhances fish immune responses (Díaz-Rosales et al. 2006, 2009; Cordero et al. 2015), improves stress tolerance (Varela et al. 2010), and modulates gut microbiota (Cordero et al. 2015), highlighting its great potential in maintaining host health.

Metagenomic analysis revealed that the Pseudomonadota possessed the most comprehensive amino acid metabolic network, including a unique serine/threonine degradation pathway that is absent in other taxa. This enrichment likely drives host amino acid metabolic changes, confirmed by our metabolomics data. Dietary supplementation of S increased essential amino acids (L-valine, L-threonine) and related metabolic intermediates. These results confirm microbiome’s pivotal role in diet-host interactions (Choi et al. 2021) and its function in regulating host metabolism (Cox et al., 2022; Lundgren et al. 2020; Roager et al. 2018; Agus et al. 2018). Multi-omics integration identified *Shewanella* as core functional taxa positively correlated with amino acids (leucine, threonine) and derivatives (e.g., 2-hydroxycinnamic acid), highlighting its role in regulating amino acid metabolism. The PLS path model further supports *Shewanella*'s key regulation of amino acid metabolism, indirectly promoting growth performance via metabolite-mediated symbiosis. Differential bacterial associations with metabolites reflect functional diversity and synergy within microbial communities, collectively shaping host physiological adaptation.

These findings hold physiological significance given essential amino acids established roles in piscine growth and health homeostasis (Shin et al. 2024; Yu et al. 2024; Yang et al. 2022). Dietary amino acid levels influence gut microbiota composition and function, inhibiting pathogen colonization (Pickard et al. 2025). Our results suggest that macroalgae supplementation of S enhances amino acids biosynthesis and derivatives accumulation. A notable example is trans-cinnamic acid, which exhibits antimicrobial activity against fish pathogens (Wang et al. 2020; Yilmaz et al. 2018) and enhances non-specific immunity (Yılmaz and Ergün, 2018)—mechanistically explaining improved growth in the macroalgae groups.

*Shewanella*’s metabolic versatility significantly contributes to the grass carp gut ecosystem. Its unique pyruvate/lactate dual fermentation capacity (Pinchuk et al. 2011) and thermostable alginate lyases-mediated polysaccharide degradation (Yagi et al. 2018) are complemented by exceptional genomic plasticity: 8.1% of coding sequences in fish-derived strains are dedicated to amino acid transport/metabolism (Chaudhary et al. 2022). This aligns with our metabolomic observations and explains its influence on host amino acid homeostasis. Crucially, a high-quality *Shewanella* MAG (C3_bin.52) revealed complete biosynthetic pathways for essential and branched-chain amino acids, confirming its genomic potential for coordinating amino acid synthesis and catabolism. *Shewanella*’s capacity to synthesize ω−3 polyunsaturated fatty acids (Moi et al. 2018) further positions it as a natural “metabolic engineering” (Liu et al. 2025) for host nutrition. These findings highlight *Shewanella* as a functionally vital taxon in mediating nutrient metabolism in aquatic species. However, the functional roles of key *Shewanella* species require further experimental validation.

The enhanced growth and immunity in grass carp may be attributed to known bioactivities of macroalgal polysaccharides. Previously reported antioxidant and immunomodulatory properties of S (Hwang et al. 2011; Li et al. 2022) and G (Bakky et al. 2023) align with our findings in S and G groups, supporting the potential of macroalgae as natural antioxidants in aquaculture. The differential regulatory effects among macroalgae species likely stem from structural variations in their bioactive components. For example, macroalgae S preferentially enriched *Shewanella* and enhanced amino acid metabolism, forming the most effective growth-promoting pathway. However, macroalgae A documented for resisting fish pathogens (Genovese et al. 2012), significantly reduced the opportunistic pathogen *Acinetobacter* and uniquely regulated tyrosine metabolism. In contrast, macroalgae G recognized for promoting gut health (Bakky et al. 2023), notably increased *Butyricicoccus*—a butyrate-producing genus linked to intestinal homeostasis and nucleotide metabolism. These functional outcomes suggest that structural differences in macroalgal polysaccharides and other bioactive compounds drive selective modulation of gut microbiota and host metabolism. Further exploration of their structure–activity relationships thus represents a compelling direction for future study.

## Conclusion

Our study demonstrates that macroalgae supplementation enhances grass carp growth by modulating gut microbiome. Macroalgae-induced restructuring of enriched *Shewanella* enhanced amino acid metabolism. Integrated multi-omics analysis revealed a diet-microbiome-metabolite axis and established *Shewanella* as a key driver of amino acid metabolism in grass carp. These mechanistic insights support developing macroalgae-based strategies to enhance nutrient metabolism in herbivorous aquaculture species, highlighting *Shewanella*’s pivotal regulatory role in amino acid metabolism.

## Supplementary Information

Supplementary Material 1.

## Data Availability

Sequencing data (16S rRNA gene and metagenomes) are available in NCBI SRA under BioProject PRJNA1293113. Metabolomics data are deposited in OMIX at CNCB-NGDC under accession OMIX010817. All data are publicly accessible.
