# Supplementary figures and images for: Dietary macroalgae enhances amino acid metabolism via intestinal Shewanella in grass carp (Ctenopharyngodon idella)
Source: Adv Biotechnol (Singap). 2025 Dec 14;3(4):36. doi: 10.1007/s44307-025-00090-8 (PMC12702836; doi:10.1007/s44307-025-00090-8)

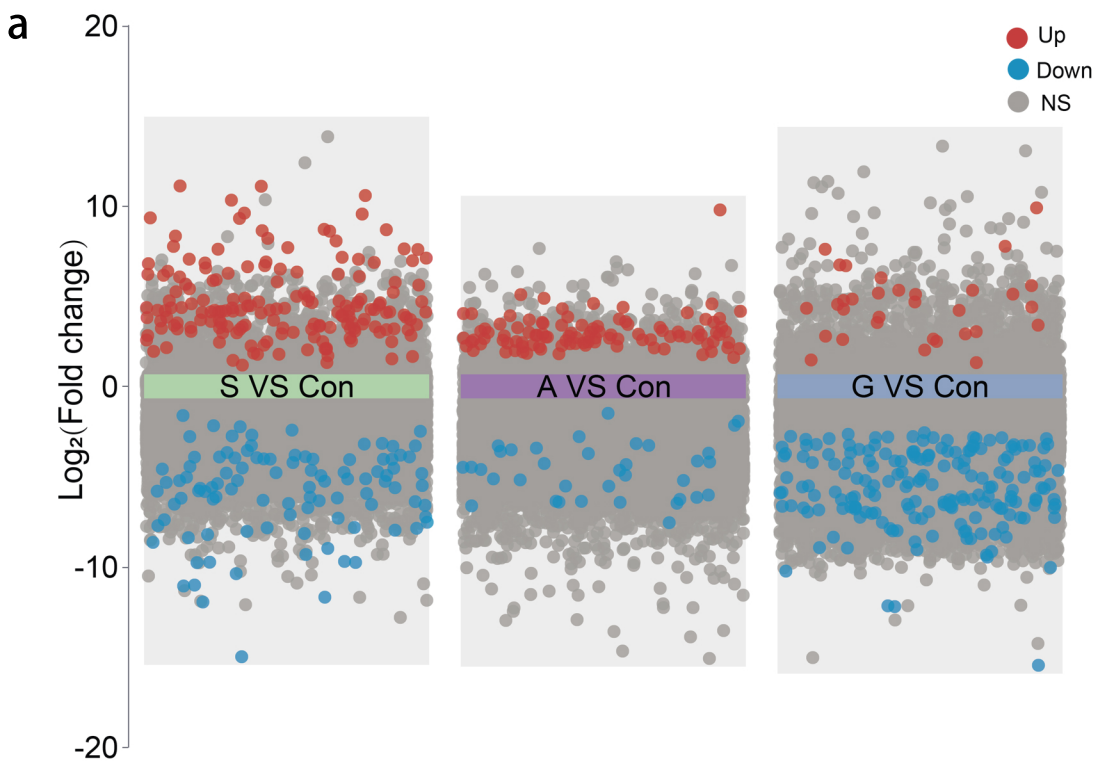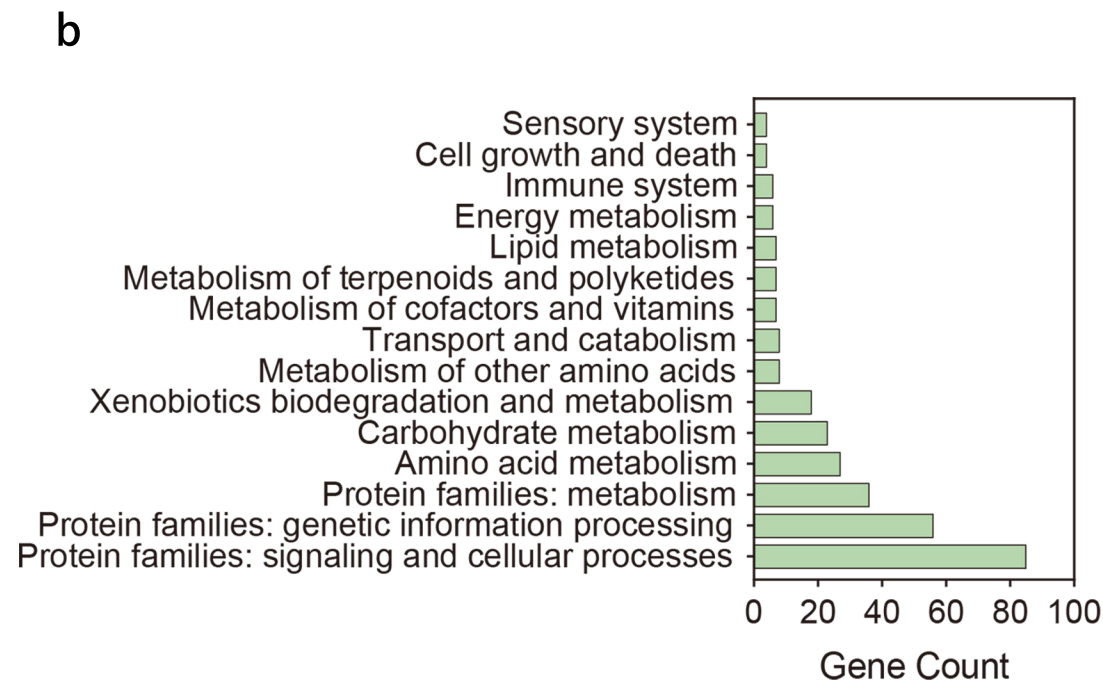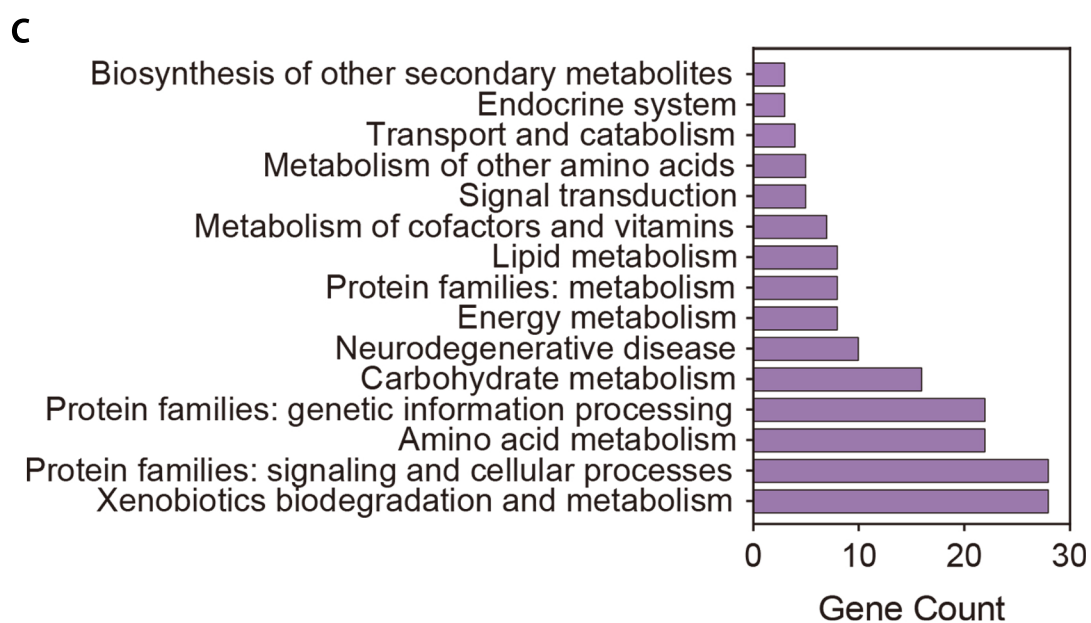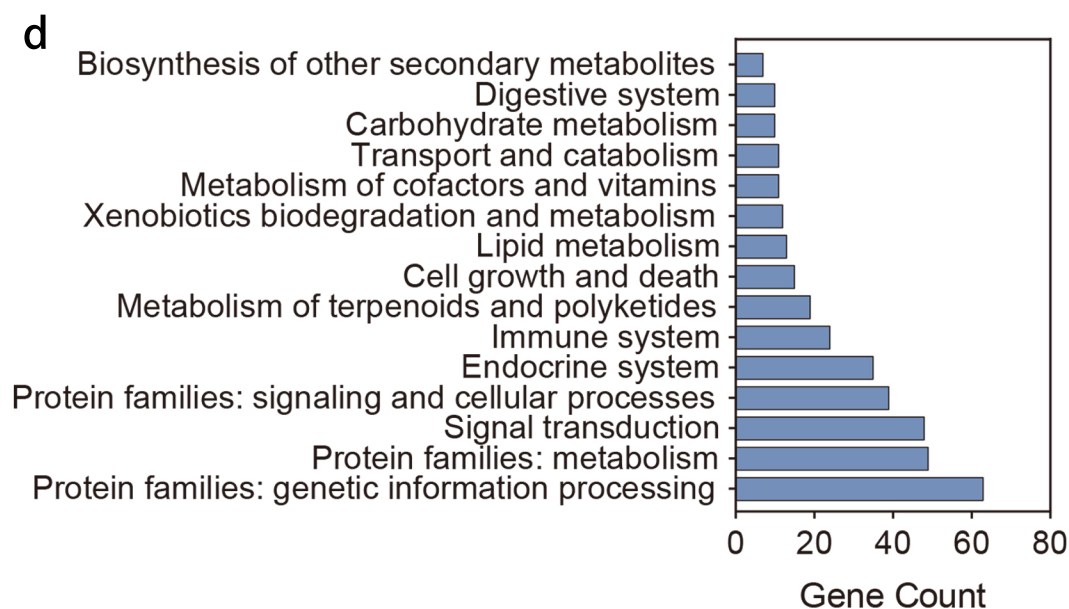

Supplement: Supplementary file 1 — Supplementary Material 1. [file 44307_2025_90_MOESM1_ESM.zip › FIGS1_ESM.pdf]

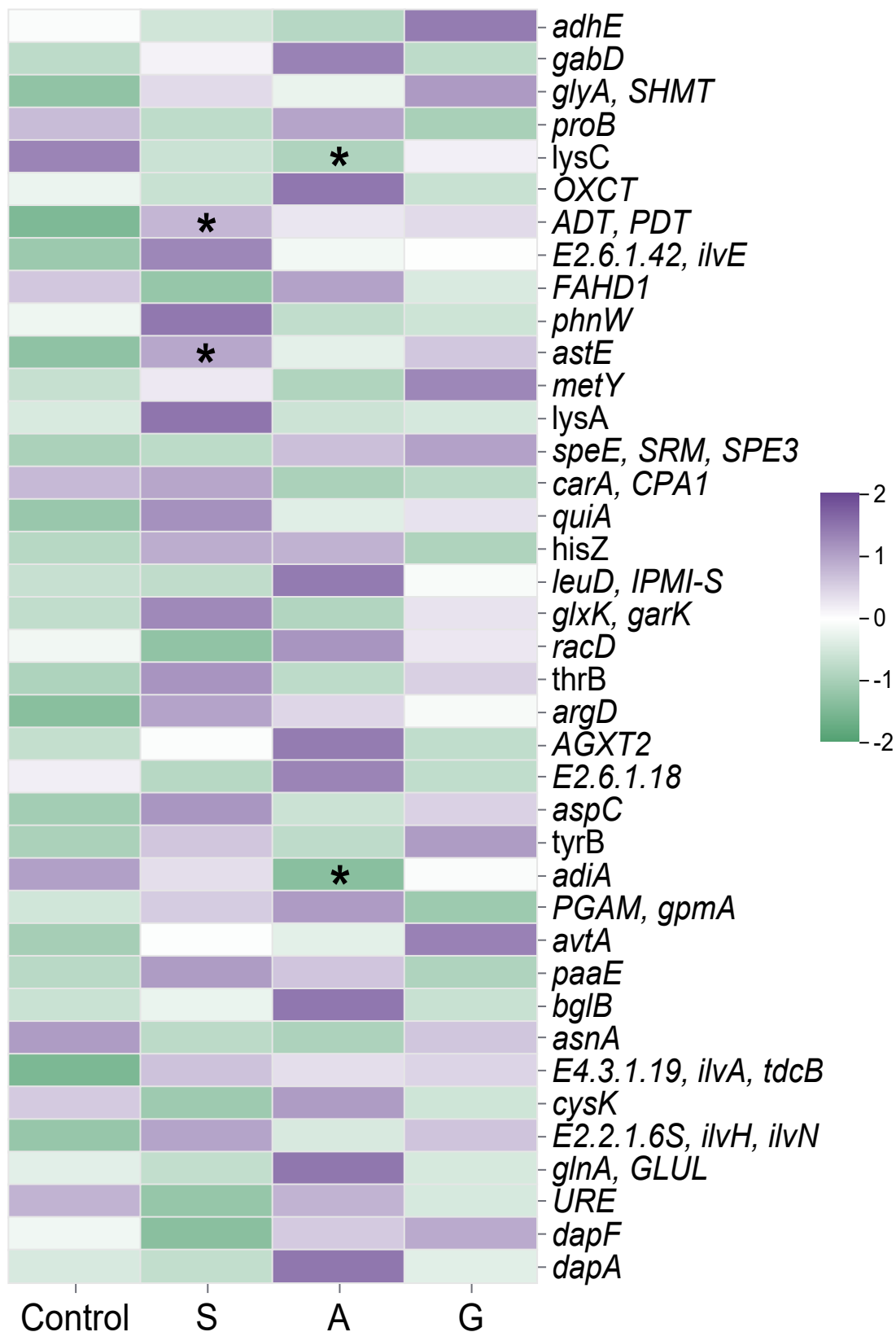

Supplement: Supplementary file 1 — Supplementary Material 1. [file 44307_2025_90_MOESM1_ESM.zip › FIGS2_ESM.pdf]

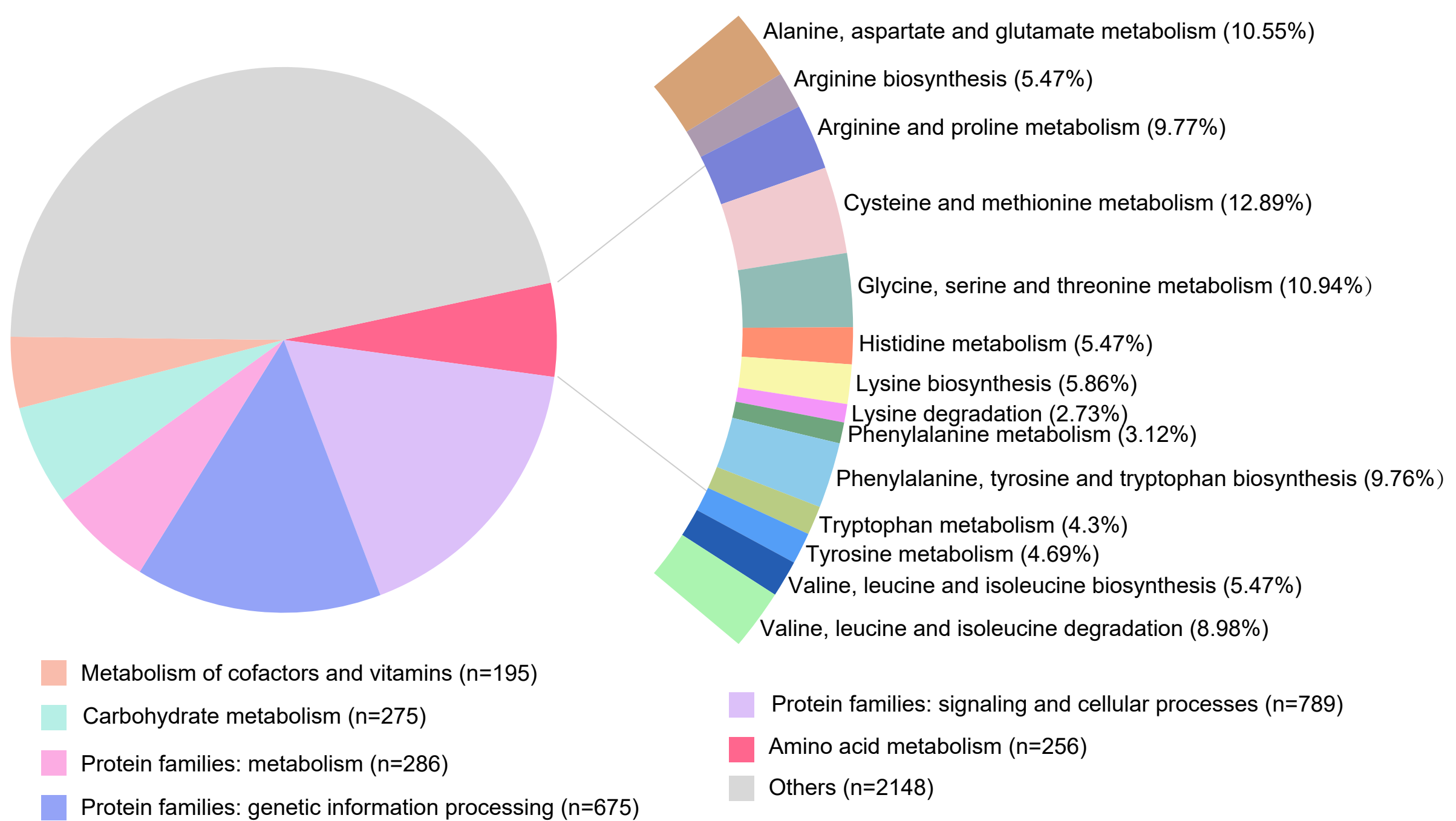

Supplement: Supplementary file 1 — Supplementary Material 1. [file 44307_2025_90_MOESM1_ESM.zip › FIGS3_ESM.pdf]
